# Complete genome sequence of *Desulfurococcus mucosus* type strain (O7/1^T^)

**DOI:** 10.4056/sigs.1644004

**Published:** 2011-04-29

**Authors:** Reinhard Wirth, Olga Chertkov, Brittany Held, Alla Lapidus, Matt Nolan, Susan Lucas, Nancy Hammon, Shweta Deshpande, Jan-Fang Cheng, Roxane Tapia, Cliff Han, Lynne Goodwin, Sam Pitluck, Konstantinos Liolios, Pagani Ioanna, Natalia Ivanova, Konstantinos Mavromatis, Natalia Mikhailova, Amrita Pati, Amy Chen, Krishna Palaniappan, Miriam Land, Loren Hauser, Yun-Juan Chang, Cynthia D. Jeffries, Yvonne Bilek, Thomas Hader, Manfred Rohde, Stefan Spring, Johannes Sikorski, Markus Göker, Tanja Woyke, James Bristow, Jonathan A. Eisen, Victor Markowitz, Philip Hugenholtz, Nikos C. Kyrpides, Hans-Peter Klenk

**Affiliations:** 1 University of Regensburg, Archaeenzentrum, Regensburg, Germany; 2 DOE Joint Genome Institute, Walnut Creek, California, USA; 3 Los Alamos National Laboratory, Bioscience Division, Los Alamos, New Mexico, USA; 4 Biological Data Management and Technology Center, Lawrence Berkeley National Laboratory, Berkeley, California, USA; 5 Oak Ridge National Laboratory, Oak Ridge, Tennessee, USA; 6 HZI – Helmholtz Centre for Infection Research, Braunschweig, Germany; 7 DSMZ - German Collection of Microorganisms and Cell Cultures GmbH, Braunschweig, Germany; 8 University of California Davis Genome Center, Davis, California, USA; 9 Australian Centre for Ecogenomics, School of Chemistry and Molecular Biosciences, The University of Queensland, Brisbane, Australia

**Keywords:** hyperthermophile, anaerobic, organotroph, sulfur respiration, spheroid-shaped, non-motile, extracellular enzymes, *Desulfurococcaceae*, GEBA

## Abstract

*Desulfurococcus mucosus* Zillig and Stetter 1983 is the type species of the genus *Desulfurococcus*, which belongs to the crenarchaeal family *Desulfurococcaceae*. The species is of interest because of its position in the tree of life, its ability for sulfur respiration, and several biotechnologically relevant thermostable and thermoactive extracellular enzymes. This is the third completed genome sequence of a member of the genus *Desulfurococcus* and already the 8^th^ sequence from a member the family *Desulfurococcaceae*. The 1,314,639 bp long genome with its 1,371 protein-coding and 50 RNA genes is a part of the *** G****enomic* *** E****ncyclopedia of* *** B****acteria and* *** A****rchaea * project.

## Introduction

Strain O7/1^T^ (= DSM 2162 = ATCC 35584 = JCM 9187) is the type strain of the species *Desulfurococcus mucosus* [1], which is the type species of its genus *Desulfurococcus*. The genus currently consists of five species with a validly published name [2]. For the genus name the Neo-Latin 'desulfo-' meaning 'desulfuricating, is used to characterize the dissimilatory sulfate-reducing feature of this spheroid-shaped 'coccus' [2]. The species epithet is derived from the Latin word 'mucosus' (slimy) [2]. Strain O7/1^T^ was isolated from an acidic hot spring in Askja, Iceland and the name of the species was effectively published by Zillig *et al*. in 1982 [1]; valid publication of the name followed in 1983 [3]. The strain was an early target for phylogenetic studies of the domain *Archaea* (at that time termed ‘*Archaebacteria*’) *via* DNA-rRNA cross-hybridizations [4,5], as well as studies on the archaeal DNA-dependent RNA polymerase structure [6] and Archaea-specific quinones [7]. Subsequently, strain O7/1^T^ was used for studies on thermostable extracellular enzymes such as proteinase [8] and pullulanase [9]. Here we present a summary classification and a set of features for *D. mucosus* strain O7/1^T^, together with a description of the complete genome sequencing and annotation.

## Classification and features

The single genomic 16S rRNA sequence of strain O7/1^T^ was compared using NCBI BLAST under default settings (e.g., considering only the high-scoring segment pairs (HSPs) from the best 250 hits) with the most recent release of the Greengenes database [10] and the relative frequencies, weighted by BLAST scores, of taxa and keywords (reduced to their stem [11]) were determined. The five most frequent genera were *Sulfolobus* (27.8%), *Aeropyrum* (11.3%), *Desulfurococcus* (11.3%), *Ignicoccus* (6.5%) and *Vulcanisaeta* (6.2%) (100 hits in total). Regarding the five hits to sequences from other members of the genus, the average identity within HSPs was 96.7%, whereas the average coverage by HSPs was 97.4%. Among all other species, the one yielding the highest score was *Desulfurococcus mobilis*, which corresponded to an identity of 100.0% and an HSP coverage of 100.0%. The highest-scoring environmental sequence was AB462558 ('Microbial production and energy source hyperthermophilic prokaryotes geothermal hot spring pool clone DDP-A01'), which showed an identity of 95.8% and a HSP coverage of 98.2%. The five most frequent keywords within the labels of environmental samples which yielded hits were 'spring' (9.2%), 'microbi' (6.8%), 'hot' (6.2%), 'nation/park/yellowston' (5.4%) and 'popul' (4.8%) (150 hits in total), indicating a good fit to the original habitat of *D. mucosus*. Environmental samples which yielded hits of a higher score than the highest scoring species were not found.

**Figure 1:**
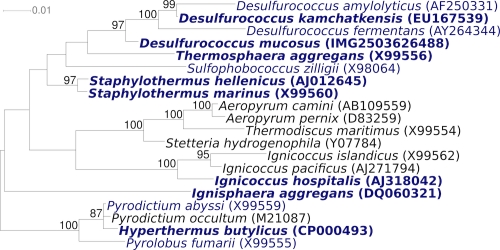
Phylogenetic tree highlighting the position of *D. mucosus* relative to the other type strains within the family *Desulfurococcaceae*. The tree was inferred from 1,334 aligned characters [12,13] of the 16S rRNA gene sequence under the maximum likelihood criterion [14] and rooted in accordance with the current taxonomy. The branches are scaled in terms of the expected number of substitutions per site. Numbers above branches are support values from 1,000 bootstrap replicates [15] if larger than 60%. Lineages with type strain genome sequencing projects registered in GOLD [16] are shown in blue, *Staphylothermus hellenicus* CP002051 and published genomes in bold [17-22].

Figure 1 shows the phylogenetic neighborhood of *D. mucosus* in a 16S rRNA based tree. A 16S rRNA reference sequence for *D. mucosus* has not been previously published.

**Figure 2:**
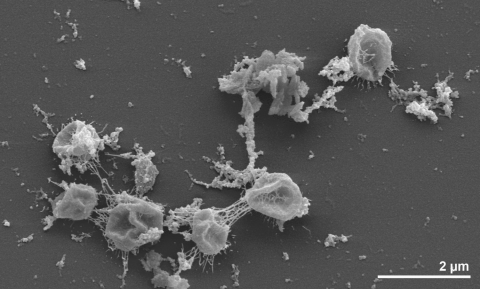
Scanning electron micrograph of *D. mucosus* strain O7/1^T^

The non-motile cells of strain O7/1^T^ are spheroid with diameters of 0.3 to 2.0 µm [1] (Figure 2), sometimes up to 10 µm [23], surrounded by a slimy mucoid layer, which covers the envelope and consists of neutral sugars and a small fraction of amino sugars [24] (Figure 2). In growing cultures, cells of strain O7/1^T^ were often found in pairs [2] (Table 1). Cells of strain O7/1^T^ can be differentiated from those of *D. mobilis*, the closest relative of *D. mucosus*, which are mobile by monopolar polytrichous flagella and devoid of the mucous polymer surrounding the *D. mucosus* cells [1,23]. Strain O7/1^T^ can utilize yeast extract and casein or its tryptic digests, but not casamino acids as the sole carbon source, by sulfur respiration with the production of H_2_S and CO_2_, or by fermentation [1]. Growing cultures synthesize a strong smelling uncharacterized product [1]. Cultures require little or no NaCl in growth media [1,23]. The temperature range for growth of strain O7/1^T^ is 76 to 93ºC, with an optimum at 85ºC [1,23]. At the optimal growth temperature, the generation time of strain O7/1^T^ was about four hours [1]. The pH range is 4.5 to 7.0, with an optimum at 6.0 [1,23]. Sugars, starch, glycogen, alcohols and intermediary metabolites are also not utilized [1]. Strain O7/1^T^ lacks an intron in the 23S RNA gene, which has been described for its close relative *D. mobilis* [35].

**Table 1:** Classification and general features of *D*. *mucosus* 07/1^T^ according to  the MIGS recommendations [25].

MIGS ID	Property	Term	Evidence code
	Current classification	Domain *Archaea*	TAS [26]
		Phylum *Crenarchaeota*	TAS [27,28]
		Class *Thermoprotei*	TAS [27,29]
		Order *Desulfurococcales*	TAS [27,30]
		Family *Desulfurococcaceae*	TAS [2,3,31]
		Genus *Desulfurococcus*	TAS [1,3,32]
		Species *Desulfurococcus mucosus*	TAS [1,3]
		Type strain O7/1	TAS [1]
	Gram stain	negative	TAS [1]
	Cell shape	spheroid, often in pairs	TAS [1]
	Motility	non-motile	TAS [1]
	Sporulation	none	NAS
	Temperature range	76°C-93°C	TAS [23]
	Optimum temperature	85°C	TAS [1,23]
	Salinity	around 0	TAS [23]
MIGS-22	Oxygen requirement	strictly anaerobic	TAS [1]
	Carbon source	yeast extract, casein or its tryptic digest	TAS [1]
	Energy metabolism	organotroph	TAS [1]
MIGS-6	Habitat	fresh water, sulfur spring	TAS [1]
MIGS-15	Biotic relationship	free living	TAS [1]
MIGS-14	Pathogenicity	none	NAS
	Biosafety level	1	TAS [33]
	Isolation	acidic hot spring	TAS [1]
MIGS-4	Geographic location	Askja, Iceland	TAS [1]
MIGS-5	Sample collection time	1981 or before	TAS [1]
MIGS-4.1	Latitude	65.05	NAS
MIGS-4.2	Longitude	-16.8	NAS
MIGS-4.3	Depth	not reported	NAS
MIGS-4.4	Altitude	approx. 1,053 m	NAS

Evidence codes - IDA: Inferred from Direct Assay (first time in publication); TAS: Traceable Author Statement (i.e., a direct report exists in the literature); NAS: Non-traceable Author Statement (i.e., not directly observed for the living, isolated sample, but based on a generally accepted property for the species, or anecdotal evidence). These evidence codes are from of the Gene Ontology project [34]. If the evidence code is IDA, then the property was directly observed by one of the authors or an expert mentioned in the acknowledgements.

### Chemotaxonomy

According to Zillig *et al*. 1982 [1], the cell envelope of the strain O7/1^T^ is flexible and probably composed of two layers of which at least the outer one appears to consist of subunits perpendicular to the surface [1]. Scarce information is available regarding the lipid composition of *D. mucosus*. The lipids in the strain O7/1^T^ are composed of phytanol and C40 polyisoprenoid dialcohols [1]. The polar lipid profile of the closely related *D. mobilis* has been studied and the structure of its three complex lipids has been elucidated in detail [36].

## Genome sequencing and annotation

### Genome project history

This organism was selected for sequencing on the basis of its phylogenetic position [37], and is part of the *** G****enomic* *** E****ncyclopedia of* *** B****acteria and* *** A****rchaea * project [38]. The genome project is deposited in the Genomes On Line Database [16] and the complete genome sequence is deposited in GenBank. Sequencing, finishing and annotation were performed by the DOE Joint Genome Institute (JGI). A summary of the project information is shown in Table 2.

**Table 2:** Genome sequencing project information

**MIGS ID**	**Property**	**Term**
MIGS-31	Finishing quality	Finished
MIGS-28	Libraries used	Three genomic libraries: one 454 pyrosequence standard library, one 454 PE library (13 kb insert size), one Illumina library
MIGS-29	Sequencing platforms	Illumina GAii, 454 GS FLX Titanium
MIGS-31.2	Sequencing coverage	75.7 × Illumina; 44.8 × pyrosequence
MIGS-30	Assemblers	Newbler version 2.5-internal-10Apr08-1-threads, Velvet, phrap
MIGS-32	Gene calling method	Prodigal 1.4, GenePRIMP
	INSDC ID	CP002363
	Genbank Date of Release	January 20, 2011
	GOLD ID	Gc02914
	NCBI project ID	48641
	Database: IMG-GEBA	2503538025
MIGS-13	Source material identifier	DSM 2162
	Project relevance	Tree of Life, GEBA

### Growth conditions and DNA isolation

*D. mucosus* strain 07/1^T^, DSM 2162, was grown anaerobically in DSMZ medium 184 (*Desulfurococcus* medium) [39] at 85°C. DNA was isolated from 0.5-1 g of cell paste using Qiagen Genomic 500 DNA kit (Qiagen 10262) following the standard protocol as recommended by the manufacturer, with no modification. DNA is available through the DNA Bank Network [40].

### Genome sequencing and assembly

The genome was sequenced using a combination of Illumina and 454 sequencing platforms. All general aspects of library construction and sequencing can be found at the JGI website [41]. Pyrosequencing reads were assembled using the Newbler assembler version 2.5-internal-10Apr08-1-threads (Roche). The initial Newbler assembly consisting of three contigs in one scaffold was converted into a phrap assembly [42] by making fake reads from the consensus, to collect the read pairs in the 454 paired end library. Illumina GAii sequencing data (99.5 Mb) were assembled with Velvet [43] and the consensus sequences were shredded into 1.5 kb overlapped fake reads and assembled together with the 454 data. The 454 draft assembly was based on 546.5 Mb 454 draft data and all of the 454 paired end data. Newbler parameters are -consed -a 50 -l 350 -g -m -ml 20. The Phred/Phrap/Consed software package [42] was used for sequence assembly and quality assessment in the subsequent finishing process. After the shotgun stage, reads were assembled with parallel phrap (High Performance Software, LLC). Possible mis-assemblies were corrected with gapResolution [41], Dupfinisher [44], or sequencing cloned bridging PCR fragments with subcloning or transposon bombing (Epicentre Biotechnologies, Madison, WI). Gaps between contigs were closed by editing in Consed, by PCR and by Bubble PCR primer walks (J.-F.Chang, unpublished). A total of 12 additional reactions were necessary to close gaps and to raise the quality of the finished sequence. Illumina reads were also used to correct potential base errors and increase consensus quality using a software Polisher developed at JGI [45]. The error rate of the completed genome sequence is less than 1 in 100,000. Together, the combination of the Illumina and 454 sequencing platforms provided 120.5 × coverage of the genome. The final assembly contained 264,988 pyrosequence and 1,310,055 Illumina reads.

### Genome annotation

Genes were identified using Prodigal [46] as part of the Oak Ridge National Laboratory genome annotation pipeline, followed by a round of manual curation using the JGI GenePRIMP pipeline [47]. The predicted CDSs were translated and used to search the National Center for Biotechnology Information (NCBI) nonredundant database, UniProt, TIGR-Fam, Pfam, PRIAM, KEGG, COG, and InterPro databases. Additional gene prediction analysis and functional annotation were performed within the Integrated Microbial Genomes - Expert Review (IMG-ER) platform [48].

## Genome properties

**Figure 3:**
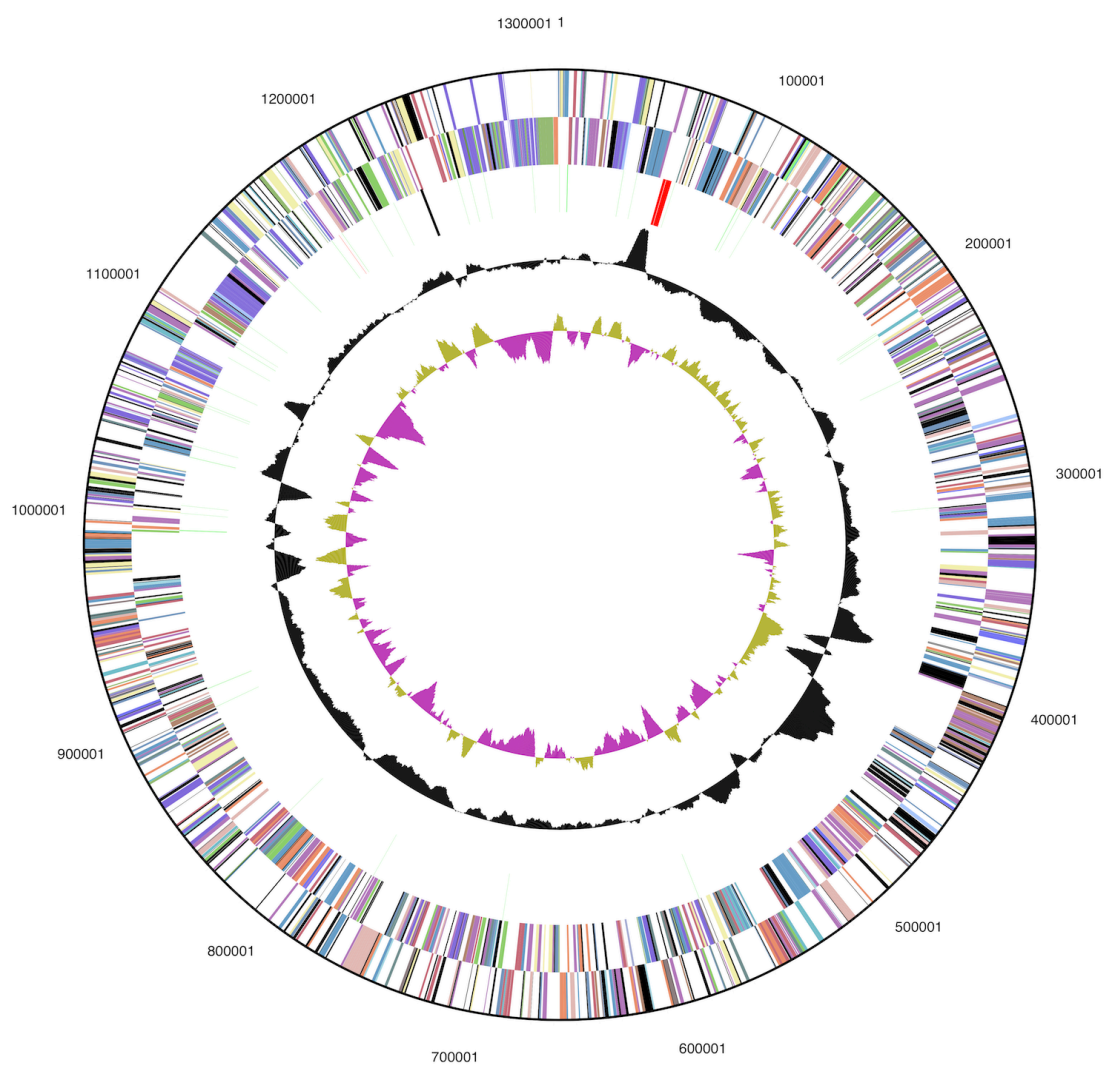
Graphical circular map of genome. From outside to the center: Genes on forward strand (color by COG categories), Genes on reverse strand (color by COG categories), RNA genes (tRNAs green, rRNAs red, other RNAs black), GC content, GC skew.

The genome consists of a 1,314,639 bp long chromosome with a G+C content of 53.1% (Table 3 and Figure 3). Of the 1,421 genes predicted, 1,371 were protein-coding genes, and 50 RNAs; 26 pseudogenes were also identified. The majority of the protein-coding genes (65.5%) were assigned with a putative function while the remaining ones were annotated as hypothetical proteins. The distribution of genes into COGs functional categories is presented in Table 4.

**Table 3:** Genome Statistics

**Attribute**	**Value**	**% of Total**
Genome size (bp)	1,314,639	100.00%
DNA coding region (bp)	1,186,810	90.28%
DNA G+C content (bp)	698,621	53.14%
Number of replicons	1	
Extrachromosomal elements	0	
Total genes	1,421	100.00%
RNA genes	50	3.52%
rRNA operons	1	
Protein-coding genes	1,371	96.48%
Pseudo genes	26	1.83%
Genes with function prediction	931	65.52%
Genes in paralog clusters	103	7.25%
Genes assigned to COGs	1,001	70.44%
Genes assigned Pfam domains	1,010	71.08%
Genes with signal peptides	146	10.27%
Genes with transmembrane helices	296	20.83%
CRISPR repeats	3	

**Table 4:** Number of genes associated with the general COG functional categories

Code	value	%age	Description
J	148	13.9	Translation, ribosomal structure and biogenesis
A	2	0.2	RNA processing and modification
K	50	4.7	Transcription
L	62	5.8	Replication, recombination and repair
B	1	0.1	Chromatin structure and dynamics
D	7	0.7	Cell cycle control, cell division, chromosome partitioning
Y	0	0.0	Nuclear structure
V	10	0.9	Defense mechanisms
T	14	1.3	Signal transduction mechanisms
M	37	3.5	Cell wall/membrane/envelope biogenesis
N	4	0.4	Cell motility
Z	0	0.0	Cytoskeleton
W	0	0.0	Extracellular structures
U	10	0.9	Intracellular trafficking, secretion, and vesicular transport
O	45	4.2	Posttranslational modification, protein turnover, chaperones
C	97	9.1	Energy production and conversion
G	52	4.9	Carbohydrate transport and metabolism
E	77	7.2	Amino acid transport and metabolism
F	39	3.7	Nucleotide transport and metabolism
H	45	4.2	Coenzyme transport and metabolism
I	14	1.3	Lipid transport and metabolism
P	81	7.6	Inorganic ion transport and metabolism
Q	3	0.3	Secondary metabolites biosynthesis, transport and catabolism
R	170	16.0	General function prediction only
S	96	9.0	Function unknown
-	420	29.6	Not in COGs
